# Tuberculosis and its associated risk factors among HIV-positive pregnant women in northwest Ethiopia: A retrospective follow-up study

**DOI:** 10.1016/j.heliyon.2023.e21382

**Published:** 2023-10-21

**Authors:** Habtamu Geremew, Anteneh Mengist Dessie, Denekew Tenaw Anley, Sefineh Fenta Feleke, Demeke Geremew

**Affiliations:** a College of Health Sciences, Oda Bultum University, Chiro, Ethiopia; b Department of Public Health, College of Health Sciences, Debre Tabor University, Debre Tabor, Ethiopia; c Department of Public Health, College of Health Sciences, Woldia University, Woldia, Ethiopia; d Department of Medical Laboratory Sciences, Immunology and Molecular Biology Unit, College of Medicine and Health Sciences, Bahir Dar University, Bahir Dar, Ethiopia

**Keywords:** Tuberculosis, Incidence, Pregnant-women, Ethiopia

## Abstract

**Background:**

People living with the human immunodeficiency virus have a higher risk of developing active tuberculosis disease. Human immunodeficiency virus infected pregnant women are at a much higher risk of getting active tuberculosis infection, partly due to immune modulation. However, very little is known about the epidemiology of tuberculosis among pregnant women infected with the virus, particularly in resource-limited settings where the burdens of these infections are substantial. Hence, this study aimed to estimate tuberculosis incidence and identify its risk factors among human immunodeficiency virus infected pregnant women in northwest Ethiopia.

**Methods:**

An institutional-based retrospective follow-up study was conducted among pregnant women who were enrolled in option B+ prevention of mother to child transmission service between June 2013 and April 2021 in Pawe district. The Kaplan–Meier survival curve and Weibull regression model were used to estimate survival probability and identify risk factors of tuberculosis, respectively. The best model between the Cox and parametric models was chosen using the Akaike and Bayesian information criteria.

**Result:**

Out of 289 human immunodeficiency virus infected pregnant women included in the final analysis, 29 (10.03 %) developed active tuberculosis. The overall incidence of tuberculosis was 17.4 per 1000 person-months of observation (95 % CI: 12.1, 25.1). Lack of isoniazid preventive therapy (AHR: 6.68, 95 % CI: 2.67, 16.7), new enrollment to care (AHR: 2.62, 95 % CI: 1.14, 6.03), under-nutrition (AHR: 5.09, 95 % CI: 2.02, 12.83), low CD4 count (AHR: 2.61, 95 % CI: 1.01, 6.78), and suboptimal antiretroviral therapy adherence (AHR: 3.17, 95 % CI: 1.46, 6.86) were predictors of tuberculosis among HIV-positive pregnant women.

**Conclusion:**

This study found a high incidence of tuberculosis among human immunodeficiency virus infected pregnant women. Thus, strengthening the provision of tuberculosis preventive therapy, reinforcing adherence support, and controlling under-nutrition should be considered to decrease the risk of tuberculosis.

## 1 Background

Tuberculosis (TB) is a contagious disease caused by the bacillus *Mycobacterium tuberculosis*; it usually affects the lungs (pulmonary TB), but it can also affect other parts of the body (Extra-pulmonary TB) [1]. In 2020, an estimated 9.9 million people were infected with tuberculosis worldwide; of all incident cases, 8 % were people infected with human immunodeficiency virus (HIV) [1]. HIV-positive people are about 18 times more likely to develop active TB disease than people without HIV; HIV and TB form a lethal combination, each speeding up the progress of the other [1,2]. HIV attacks the immune system, exposing infected individuals to co-infection with TB or the progression of latent TB [3,4]. Similarly, TB infection increases viral replication, thereby promoting its progress to advanced stages [4].

About 1.1 million people are living with TB and HIV worldwide, of which 80 % are in sub-Saharan Africa [5]. Ethiopia is one of the 30 countries with the highest burden of both TB and TB-HIV co-infections worldwide [6]. Several studies were undertaken worldwide to assess the magnitude of TB among HIV-positive patients. For instance, studies conducted in Cambodia and Tanzania reported TB incidences of 3.9 and 4.4 per 100 person-years, respectively [7,8]. In Ethiopia, the incidence of TB among adult HIV patients receiving antiretroviral therapy (ART) ranges from 3.1 to 8.79 per 100 person-years [9,10]. However, the equivalent figure for HIV-positive pregnant women receiving lifelong ART remains unknown in the country.

The risk and consequences of active TB infection are much higher among women infected with HIV [[11], [12], [13]]. Pregnancy related immunological changes also increase women's vulnerability to new infection and activation of latent TB infection [14]. Hormonal change during pregnancy leads to reduced production of CD4 Th1 cells, which are the major actors of our immune defense against *Mycobacterium tuberculosis* [4]. Thus, pregnant women are at high risk of TB infection, especially if they are also HIV positive [[15], [16], [17]]. TB rates are up to ten times higher in HIV-positive pregnant women than in HIV-negative pregnant women [2,12].

Tuberculosis is the leading cause of death among HIV-positive people [1]. For women, tuberculosis can have serious consequences, especially during their reproductive years and during pregnancy. TB increases the risk of maternal and infant mortality by nearly 300 % in HIV-positive pregnant women, accounting for 15–34 % of indirect causes of obstetric mortality [12,18,19]. TB-HIV co-infection is associated with higher risks for several obstetric complications [19,20]. Furthermore, TB in HIV-positive mothers raises the risk of vertical HIV transmission to the unborn child [12,21]. Thus, maternal TB and HIV in pregnant women expose the mother and the fetus to an increased risk and complications of these two serious infections [18].

Measures like integration of TB-HIV programs, provision of isoniazid preventive therapy, and routine TB screening in HIV-infected individuals are being implemented to mitigate the burden of TB among HIV-infected people in Ethiopia [22]. Besides, universal ART treatment for all HIV-positive pregnant and breastfeeding women has also been implemented since 2013 [23]. Nevertheless, despite the known synergy between TB and HIV, there is a paucity of evidence on the epidemiology of tuberculosis infection among pregnant women infected with HIV after the inauguration of option B+ lifelong ART, particularly in resource-limited settings where the burdens of these infections are substantial. Thus, this study was conducted to assess the incidence of tuberculosis and its associated risk factors among HIV-positive pregnant women in Pawe district, northwest Ethiopia, after the initiation of option B+ lifelong ART.

## 2 Methods and materials

### 2.1 Study design, area, and period

An institutional-based retrospective follow-up study was conducted in Pawe General Hospital and Felege Selam Health Center, which were the only health facilities that provide lifelong ART service in Pawe District, northwest Ethiopia. The study was conducted retrospectively using data recorded from June 2013 through April 2021, which denotes the period after the inauguration of option B+ lifelong ART in Ethiopia.

### 2.2 Population

All pregnant women who were enrolled to lifelong ART in Pawe District were the source population for this study. Whereas, all pregnant women who were enrolled to lifelong ART in Pawe General Hospital and Felege Selam Health Center between June 2013 and April 2021 constitute our study population.

### 2.3 Inclusion and exclusion criteria

All pregnant women who were enrolled to lifelong ART during the study period and had at least one month of ART follow-up were included in the study. However, women with incomplete records in regard to variables of interest and women who were diagnosed with TB or on anti-TB at enrollment were excluded from the study.

### 2.4 Sample size and sampling procedure

Although all women who fulfilled the inclusion criteria were included in the study, the adequacy of the sample size was checked using the “stpower” command of STATA, considering a 95 % confidence interval, 80 % power, 15 % withdrawal probability, and hazard ratios from previous reports [24,25]. Accordingly, the largest calculated sample size was 202; however, all women's records that fulfilled the inclusion criteria (289) were included to increase the power of the study.

### 2.5 Variables of the study

The outcome variable of the study was the incidence rate of TB co-infection among HIV-positive pregnant women. Independent variables include socio-demographic characteristics, clinical characteristics, and treatment-related characteristics of women.

### 2.6 Operational definitions

Event: occurrence of TB during the follow-up time as ascertained by reviewing women's records.

Censored: if a woman delivered, aborted, lost follow-up, transferred out to another facility, or was on ART but didn't develop TB by the end of the follow-up.

Incident tuberculosis: occurrence of TB in an HIV-positive pregnant woman who was TB-free at enrollment to the study, as confirmed by reviewing the woman's records. The diagnosis of TB was made using bacteriological methods like smear microscopy, Xpert MTB/RIF assay, or clinically based on the clinician's decision.

Rapid ART initiation: women who started ART within seven days of HIV diagnosis [26].

Delayed ART initiation: women who started ART at any time after seven days of HIV diagnosis [26].

Good ART adherence: drug adherence of 95 %, or ≤2 missed drug doses of 30 doses, or <3 missed drug doses of 60 doses [27].

Fair ART adherence: drug adherence of 85–94 %, or 3–5 missed drug doses of 30 doses, or 4–9 missed drug doses of 60 doses [27].

Poor ART adherence: drug adherence of <85 %, or ≥6 missed drug doses of 30 doses, or >9 missed drug doses of 60 doses [27].

Sub-optimal ART adherence: if the woman reported either poor or fair adherence.

Incomplete record: records with missing or inconsistent information regarding variables of interest like event/outcome, date event occurred, or date of enrollment to lifelong ART.

### 2.7 Data collection, management, and analyses

Secondary data were extracted from the prevention of mother to child transmission (PMTCT) register, ART intake form, ART follow-up form, and patient card by three trained midwives working at the PMTCT unit, and they were supervised by a senior trained B.Sc. midwife.

The data were entered using Epidata version 3.1 and then exported to STATA version 14 for cleaning, coding, and further statistical analysis. The survival experience of women was assessed using the Kaplan-Meier survivor function, and survival experience between different groups of categorical independent variables was compared using the log-rank test. Both graphical and statistical methods were employed to check for proportional hazard assumptions. Multicollinearity between independent variables was assessed using the variance inflation factor (VIF) [28].

Factors significantly associated with TB in the univariate models at a P value less than 0.2 were included in the multivariable survival model. The semi-parametric Cox proportional hazard model and parametric survival models were fitted to identify independent predictors of tuberculosis. A more parsimonious hazard model was chosen using the Akaike information criterion (AIC) and Bayesian information criterion (BIC). The association between predictor variables and TB was summarized using the adjusted hazard ratio (AHR) with its respective 95 % confidence interval (CI) and a significant statistical association was declared at a p-value less than 0.05.

## 3 Results

### 3.1 Participants

Of the 347 pregnant women enrolled for ART care in the aforementioned facilities between June 2013 and April 2021, 289 were included in the final analysis. Thirty-one had TB at enrollment, 21 records were incomplete, and six were not eligible (didn't have a one-month follow-up).

### 3.2 Socio-demographic characteristics of study participants

The age of respondents ranged from 15 to 43 years, with a mean (standard deviation (SD)) age of 28.7 (5.2) years. The majority (62.98 %) of respondents were urban dwellers. Most (38.41 %) of the women had no formal education (Table 1).

**Table 1:** Socio-demographic characteristics of HIV-positive pregnant women on ART care in Pawe District, northwest Ethiopia.

Variable	Category	Frequency	Percentage
Age	15–24	62	21.45
	25–34	181	62.63
	35–44	46	15.92
Residence	Urban	182	62.98
	Rural	107	37.02
Woman reside within the catchment area	Yes	249	86.16
	No	40	13.84
Marital status	Never married	24	8.32
	Married	230	79.58
	Divorced/Widowed	35	12.10
Educational status	No formal education	111	38.41
	Primary	83	28.72
	Secondary	74	25.61
	Tertiary	21	7.27
Occupation	Housewife	195	67.47
	Governmental employee	32	11.07
	Farmer	19	6.57
	Merchant	16	5.54
	Others^a^	27	9.35
Religion	Orthodox	233	80.62
	Muslim	34	11.76
	Protestant/Catholic	22	7.62

**Key:**^a^Student, Daily laborer, or Commercial sex worker.

### 3.3 Clinical characteristics of study participants at enrolment

At enrolment, the majority (88.93 %) of the study participants were classified as WHO clinical stage-Ι followed by stage-Ⅱ 35 (9.34 %). Most (93.43 %) of the participants had CD4 counts greater than or equal to 200 cells per microliter of blood (Table 2).

**Table 2:** Table 2: Clinical characteristics of HIV-positive pregnant women on ART care in Pawe District, northwest Ethiopia.

Variable	Category	Frequency	Percentage
Partner HIV status	Positive	150	51.90
	Negative	58	20.07
	Unknown	81	28.03
WHO stage at enrollment	One	257	88.93
	Two	27	9.34
	Three	5	1.73
Hematocrit	≥33 %	241	83.39
	<33 %	48	16.61
MUAC	≥23 cm	200	69.20
	<23 cm	89	30.80
CD4	≥200	270	93.43
	<200	19	6.57
Opportunistic infection	Yes	34	11.76
	No	255	88.24
Disclosure status	Yes	254	87.89
	No	35	12.11
Functional status	Working	286	98.96
	Ambulatory	3	1.04
Gestational age	1st trimester	239	82.70
	2nd trimester	31	10.73
	3rd trimester	19	6.57

**Keys:** HIV, human immunodeficiency virus; MUAC, mid-upper arm circumference; CD4: cluster of differentiation 4.

### 3.4 Treatment-related characteristics of study participants

More than half (56.75 %) of the respondents experienced delayed ART initiation. Most (78.89 %) of women were on ART before enrollment to PMTCT, whereas the remaining were newly enrolled to care (Table 3).

**Table 3:** Treatment-related characteristics of HIV-positive pregnant women on ART care in Pawe District, northwest Ethiopia.

Variable	Category	Frequency	Percentage
Time of ART initiation	Rapid	125	43.25
	Delayed	164	56.75
Enrollment type to PMTCT	From ART	228	78.89
	New	61	21.11
Facility treatment attended	Hospital	251	86.85
	Health center	38	13.15
Adherence to ART	Good	257	88.93
	Fair	17	5.88
	Poor	15	5.19
Initial ART regimen	AZT+3 TC + NVP	46	15.92
	AZT+3 TC + EFV	24	8.30
	TDF+3 TC + EFV	183	63.32
	TDF + FTC + NVP	31	10.73
	TDF+3 TC + DTG	5	1.73
Drug side effect	Yes	17	5.88
	No	272	94.12
Maternal CPT	Yes	52	17.99
	No	237	82.01
Maternal INH	Yes	266	92.04
	No	23	7.96
Past TB history	Yes	5	1.73
	No	284	98.27

**Keys**: 3 TC, lamivudine; ART, antiretroviral therapy; AZT, azidothymidine; CPT, cotrimoxazole prevention therapy; DTG, dolutegravir; EFV, efavirenz; FTC, emtricitabine; INH, isoniazid; NVP, nevirapine; PMTCT, prevention of mother to child transmission; TB, tuberculosis; TDF, tenofovir disoproxil fumarate.

### 3.5 Incidence of tuberculosis among pregnant women

**Fig. 1:**
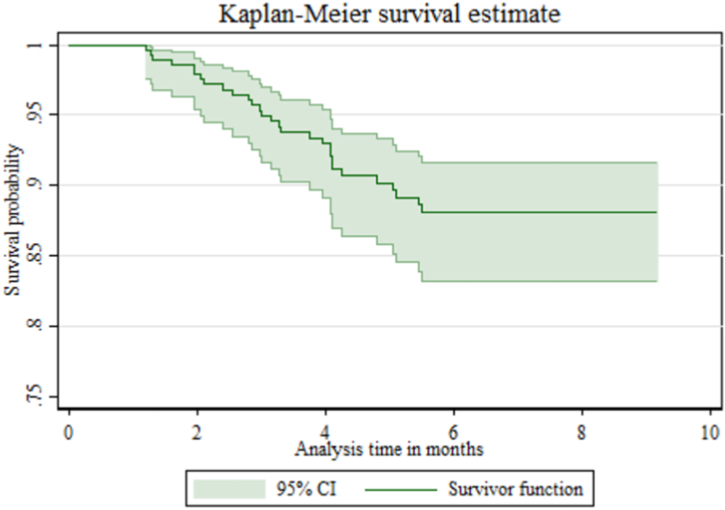
Overall Kaplan-Meier survival estimates of TB-free survival time of HIV-positive pregnant women on ART care in Pawe District, northwest Ethiopia.

In this study, a total of 289 HIV-positive pregnant women were followed for a different period, contributing to a cohort of 1664.43 person-months of observation. The minimum, maximum, and median time of follow-up were 1.17, 9.17, and 6.43 months, respectively. Out of 289 women followed during this study, 29 developed active TB disease (22 developed pulmonary TB while 7 had extra-pulmonary TB), 143 gave birth, 31 encountered abortion, 27 were transferred out to other facilities, 36 were lost to follow-up, and 23 were on ART but didn't develop TB. The overall incidence of TB was 17.4 per 1000 person-months of observation (95 % CI: 12.1, 25.1) (Fig. 1).

### 3.6 Risk factors of tuberculosis

Before fitting the final model, the proportional hazard assumption was checked. Consequently, the Schoenfeld global test indicated the assumption was met (P = 0.95). The Cox proportional hazard model and parametric models were fitted to identify determinants of TB, and the most parsimonious model (Weibull) was chosen based on the Akaike and Bayesian information criteria (Table 4).

**Table 4:** Summary of model comparison between Cox proportional hazard model and parametric survival regression models using AIC and BIC.

Parameter	Cox	Exponential	Weibull	Gompertz	Lognormal	Llogistic	Ggamma
AIC	263.0154	178.0825	173.2094	178.5275	174.1749	173.6453	174.9074
BIC	285.0139	203.7475	202.5408	207.859	203.5064	202.9767	207.9052

**Keys:** AIC, Akaike information criterion; BIC, Bayesian information criterion; Llogistic, Log logistic; Ggamma; Generalized gamma.

In the bi-variable analysis, eleven variables (occupation, WHO clinical stage, nutritional status, CD4 count, opportunistic infection, disclosure status, enrollment type, ART drug adherence, drug side effect, anemia, and isoniazid preventive therapy) were found to be associated at a *P*-value of 0.2. However, after adjusting for confounding, only five variables (nutritional status, CD4 count, enrollment type, ART drug adherence, and isoniazid preventive therapy) were found to have a statistically significant association with TB incidence at a *P*-value of 0.05.

According to the multivariable Weibull regression model, women who didn't take isoniazid preventive therapy had a 6.68 (95 % CI: 2.67, 16.7) times higher risk of TB when compared to those who did. The risk of TB was 2.62 (95 % CI: 1.14, 6.03) times higher among newly enrolled women than among women who were previously on ART. Similarly, undernourished women had a 5.09 (95 % CI: 2.02, 12.83) times higher risk of TB as compared to well-nourished women. Likewise, pregnant women with a CD4 count below 200 cells per microliter of blood had a 2.61 (95 % CI: 1.01, 6.78) times higher risk of TB than women with a CD4 count of 200 or more. Furthermore, the hazard of developing TB was 3.17 (95 % CI: 1.46, 6.86) times higher among pregnant women who had poor and/or fair levels of ART adherence as compared to those who had good adherence (Table 5).

**Table 5:** Risk factors of TB among HIV-positive pregnant women on ART care in Pawe District, northwest Ethiopia.

Variable	Category	Survival status		CHR	AHR (95 % CI)
		Censored	Event		
Maternal INH	Yes	245	21	1	1
	No	15	8	4.61	6.68 (2.67, 16.7)*
Hematocrit	≥33 %	225	16	1	1
	<33 %	35	13	5.03	2.23 (0.86, 5.74)
MUAC	≥23 cm	192	8	1	1
	<23 cm	68	21	6.83	5.09 (2.02, 12.83)*
CD4	<200	11	8	6.26	2.61 (1.01, 6.78)*
	≥200	249	21	1	1
Adherence to ART	Optimal	241	16	1	1
	Suboptimal	19	13	8.43	3.17 (1.46, 6.86)*
Enrollment type	From ART	212	16	1	1
	New	48	13	4.6	2.62 (1.14, 6.03)*

**Keys:** *significant at p-value <0.05, AHR, adjusted hazard ratio; CHR, crude hazard ratio; CI, confidence interval; INH, isoniazid; MUAC, mid-upper arm circumference; CD4, cluster of differentiation 4; ART, antiretroviral therapy.

## 4 Discussion

Determining the epidemiology and clinical features of TB co-infection among HIV-infected pregnant women is essential for addressing the increased risks of adverse perinatal outcomes. As a result, this study was conducted to determine the incidence of tuberculosis and its predictors among HIV-positive pregnant women in northwest Ethiopia. Consequently, the overall incidence of TB was 17.4 per 1000 person-months of observation (this is equivalent to 20.9 per 100 person-years of observation). This finding is higher than previous reports from Debre Markos, northwest Ethiopia: 6.5 per 100 person-years of observation [24], southern Ethiopia: 8.79 per 100 person-years [10], central Ethiopia: 3.59 per 100 person-years [25], northeast Ethiopia: 8.6 per 100 person-years of observation [29]. This might be due to the high rate of non-adherence to lifelong ART care in the current study area [30].

Our TB incidence estimate was also higher than reports from other countries such as Tanzania: 4.4 per 100 person-years of observation [8], Nigeria: 0.57 per 100 person-years [31], South Africa: 11.27 per 1000 person-years of observation [32], and Cambodia: 3.9 per 100 patient-years of observation [7]. This variation in the incidence of TB could be attributed to differences in the study population. The previous studies were conducted among HIV-positive adults, whereas the current study was conducted among HIV-positive pregnant women, and it is evidenced elsewhere that pregnancy increases the risk of TB, particularly when they are HIV infected [11,13,15,17].

Moreover, the present study also identified different risk factors for TB. Accordingly, pregnant women who had not taken isoniazid (INH) were 6.68 times more likely to develop TB as compared to those who took INH. This association was also clearly demonstrated by previous studies [24,33,34] and underlines the essential role of INH in reducing the risk of TB among people living with HIV [35,36]. Similarly, the risk of TB was 2.62 times higher among newly enrolled women than among women who were previously on ART. This could be partly due to the immune reconstitution inflammatory syndrome, which is common in the early stages of ART [37,38]. Poor adherence to ART, which is usual among newly enrolled women, could also be another reason [39].

Likewise, undernourished women had a 5.09-fold higher TB risk as compared to well-nourished women. This association was also disclosed in other studies [7,29]. The possible explanation could be due to the further deterioration of women's immunity due to under-nutrition, which is already suppressed by the virus, thus making women more susceptible to opportunistic infections, including TB [40,41]. Consistent with other studies [34,42,43], pregnant women who had a CD4 count below 200 cells per microliter of blood had a 2.61 times higher risk of TB than women who had CD4 counts greater than 200 cells per microliter of blood. This could be partly because CD4 counts are the best measure of women's immune and clinical status. Hence, a lower CD4 count indicates substantial immunodeficiency and increased vulnerability to infection [44,45].

Furthermore, as indicated by existing evidence [25,43], the hazard of TB was 3.17 times higher among women with suboptimal ART adherence when compared to women with good adherence to ART. This might be due to the fact that the protective effect of ART might be lost in suboptimal adherence. Besides, women with poor adherence might have poor viral suppression and incompetent immunity, hence exposing them to infection [46,47]. Hence, indicates the need for strengthened TB-HIV collaborative activities in Ethiopia.

This study has the following strengths: firstly, it demonstrates practicable findings that are corroborated by existing evidence. Secondly, our study is the first of its kind in Ethiopia, especially in the underserved regions of the country. Hence, it can be used as baseline data for further inquiries. However, the interpretation of the findings should be done with due consideration of the following limitations: firstly, as it was based on a retrospective analysis of secondary data, potentially important variables like socioeconomic status and housing conditions were not obtained from the records. Secondly, the exclusion of incomplete records and being facility-based might introduce selection bias and affect the representativeness of the study. Lastly, the study focused only on HIV-positive pregnant women enrolled in PMTCT care; hence, the findings might not be mirrored for non-pregnant or HIV-negative women or those who are not enrolled in PMTCT care.

## 5 Conclusions

This study found a high TB incidence. Lack of isoniazid preventive therapy, under-nutrition, low CD4 count, suboptimal ART adherence, and new enrollment to ART were the significant risk factors for TB incidence. Thus, healthcare workers should provide an individualized patient care considering the identified risk factors. Besides, higher officials should emphasize strengthening the provision of tuberculosis preventive therapy, reinforcing adherence support, and controlling under-nutrition to decrease the risk of tuberculosis incidence among HIV-positive pregnant women.

## Ethics considerations

Ethical clearance was granted by 10.13039/501100021567Debre Markos University College of Health Science ethical review committee (reference No: HSC/R/C/Ser/Co/240/11/13). Permission for data collection was obtained from Pawe General Hospital and Felege Selam Health Center. Furthermore, confidentiality of the extracted information was assured by not recording patients’ personal identifiers and using the abstracted data only for the purpose of this research.

## Availability of data

Data included in article/supp. material/referenced in article.

## Funding

No fund was obtained for this study.

## Additional information

No additional information is available for this paper.

## CRediT authorship contribution statement

**Habtamu Geremew:** Writing – review & editing, Writing – original draft, Visualization, Validation, Supervision, Software, Resources, Project administration, Methodology, Investigation, Formal analysis, Data curation, Conceptualization. **Anteneh Mengist Dessie:** Writing – review & editing, Visualization, Resources, Project administration, Methodology, Investigation. **Denekew Tenaw Anley:** Writing – review & editing, Validation, Resources, Methodology, Investigation. **Sefineh Fenta Feleke:** Writing – review & editing, Validation, Resources, Project administration, Methodology, Investigation. **Demeke Geremew:** Writing – review & editing, Supervision, Software, Methodology, Investigation, Formal analysis, Data curation, Conceptualization.

## Declaration of competing interest

The authors declare that they have no known competing financial interests or personal relationships that could have appeared to influence the work reported in this paper.
